# Novel ITS1 Fungal Primers for Characterization of the Mycobiome

**DOI:** 10.1128/mSphere.00488-17

**Published:** 2017-12-13

**Authors:** Mykhaylo Usyk, Christine P. Zolnik, Hitesh Patel, Michael H. Levi, Robert D. Burk

**Affiliations:** a Department of Pediatrics, Albert Einstein College of Medicine, Bronx, New York, USA; b Department of Biology, Long Island University, Brooklyn, New York, USA; c Department of Pathology, Montefiore Medical Center, Bronx, New York, USA; d Department of Pathology, Albert Einstein College of Medicine, Bronx, New York, USA; e Departments of Obstetrics & Gynecology and Women’s Health, Epidemiology and Population Health, and Microbiology & Immunology, Albert Einstein College of Medicine, Bronx, New York, USA; Carnegie Mellon University

**Keywords:** ITS1, yeast, fungi, mycobiome, oral, primer design

## Abstract

Studies of the human microbiome frequently omit characterization of fungal communities (the mycobiome), which limits our ability to investigate how fungal communities influence human health. The internal transcribed spacer 1 (ITS1) region of the eukaryotic ribosomal cluster has features allowing for wide taxonomic coverage and has been recognized as a suitable barcode region for species-level identification of fungal organisms. We developed custom ITS1 primer sets using iterative alignment refinement. Primer performance was evaluated using *in silico* testing and experimental testing of fungal cultures and human samples. Using an expanded novel reference database, SIS (18S-ITS1-5.8S), the newly designed primers showed an average *in silico* taxonomic coverage of 79.9% ± 7.1% compared to a coverage of 44.6% ± 13.2% using previously published primers (*P* = 0.05). The newly described primer sets recovered an average of 21,830 ± 225 fungal reads from fungal isolate culture samples, whereas the previously published primers had an average of 3,305 ± 1,621 reads (*P* = 0.03). Of note was an increase in the taxonomic coverage of the *Candida* genus, which went from a mean coverage of 59.5% ± 13% to 100.0% ± 0.0% (*P* = 0.0015) comparing the previously described primers to the new primers, respectively. The newly developed ITS1 primer sets significantly improve general taxonomic coverage of fungal communities infecting humans and increased read depth by an order of magnitude over the best-performing published primer set tested. The overall best-performing primer pair in terms of taxonomic coverage and read recovery, ITS1-30F/ITS1-217R, will aid in advancing research in the area of the human mycobiome.

**IMPORTANCE** The mycobiome constitutes all the fungal organisms within an environment or biological niche. The fungi are eukaryotes, are extremely heterogeneous, and include yeasts and molds that colonize humans as part of the microbiome. In addition, fungi can also infect humans and cause disease. Characterization of the bacterial component of the microbiome was revolutionized by 16S rRNA gene fragment amplification, next-generation sequencing technologies, and bioinformatics pipelines. Characterization of the mycobiome has often not been included in microbiome studies because of limitations in amplification systems. This report revisited the selection of PCR primers that amplify the fungal ITS1 region. We have identified primers with superior identification of fungi present in the database. We have compared the new primer sets against those previously used in the literature and show a significant improvement in read count and taxon identification. These primers should facilitate the study of fungi in human physiology and disease states.

## Abstract (precis)

The mycobiome constitutes all the fungal organisms within an environment or biological niche. The fungi are eukaryotes, are extremely heterogeneous, and include yeasts and molds that colonize humans as part of the microbiome. In addition, fungi can also infect humans and cause disease. Characterization of the bacterial component of the microbiome was revolutionized by 16S rRNA gene fragment amplification, next-generation sequencing technologies, and bioinformatics pipelines. Characterization of the mycobiome has often not been included in microbiome studies because of limitations in amplification systems. This report revisited the selection of PCR primers that amplify the fungal ITS1 region. We have identified primers with superior identification of fungi present in the database. We have compared the new primer sets against those previously used in the literature and show a significant improvement in read count and taxon identification. These primers should facilitate the study of fungi in human physiology and disease states.

## INTRODUCTION

As innovations in the field of next-generation sequencing (NGS) progress and high-throughput bioinformatic analyses become more prevalent, the microbiome has emerged as a field of increasing importance. Microbiome studies have primarily focused on the identification of significant bacterial taxa or community states in human- and animal-pathogenic conditions because of the ease of community characterization by sequencing PCR-amplified fragments of the prokaryotic 16S rRNA gene (1). This gene is ideal for bacterial identification due to its universal presence in prokaryotes, homologous structure, and evolutionary relatedness allowing taxonomic reconstruction. Although fungi have been shown to be important in human health due in part to their ability to secrete highly active metabolites that are directly involved in pathogenesis (2) and indirectly through modulation of the microbiome (3), the study of pathogenic and nonpathogenic fungi and fungal communities in general has lagged behind studies of bacteria (4, 5).

**FIG 1:**
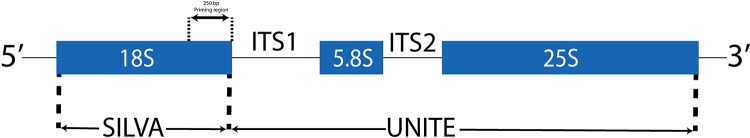
ITS rRNA gene locus. Schematic of the eukaryotic ribosomal gene cluster. The SILVA database contains sequences of the 18S gene, while the UNITE database contains sequences from the ITS1-5.8S-ITS2-25S rRNA gene cluster (not to scale). For development of our custom primers, we created a merged SILVA and UNITE database to simulate the 18S-ITS1-5.8S region. A 250-bp region at the 3′ end of the 18S gene was individually isolated when designing the forward primers.

Unlike bacteria, fungal evolution and morphological diversity are not intimately associated with single genetic markers. The morphological system that has primarily been used to classify fungi presents a barrier for researchers by including multiple potential species names for the same organism. For example, morphological differences present throughout the various reproductive stages of fungi (i.e., anamorph and telemorph) (6) may result in multiple taxonomic classifications for the same organism. In contrast, molecular methods that target specific genetic regions such as cytochrome *c* oxidase subunit 1 (CO1) (7) and the internal transcribed spacers (ITSs) (see Fig. 1) of the eukaryotic ribosomal cluster (8), provide a new paradigm for fungal taxonomy. Placement of the same organism into multiple groups due to morphological characterization confounds molecular classification, especially when there is significant genetic diversity in the organism of interest. Investigators are therefore limited to the use of genetic markers that are informative for species-level identification, but unfortunately lack the ability to perform phylogenetic reconstruction given the complex evolutionary history of fungi (4). A currently proposed barcode DNA region for fungal community identification is the ITS1 region, which allows for species-level resolution in a large number of fungi and provides amplicon sizes suitable for current short read NGS platforms like Illumina (9).

Despite the availability of a suitable DNA region for fungal community analyses, studies of medically relevant fungi use previously developed, “universal” ITS1 primers that are limited by taxonomic bias as they were developed based on small subset of soil fungi available at the time of their creation and commonly generate a low number of sequence reads (10–14). The latter is especially important because community-based analyses suffer when a sample does not achieve a minimum read threshold necessary to characterize the community—a threshold readily identifiable through rarefaction analysis (15). The present study utilizes bioinformatics and an iterative approach to identify fungal sequences for primer design for broad taxonomic coverage using the ITS1 barcode region. This study further expands on the currently available tools by creating a database of contiguous 18S-ITS1-5.8S (SIS) sequences, which are omitted from assembled fungal reference genomes and makes it possible to bioinformatically evaluate primer pairs. In addition to *in silico* evaluation against the SIS database, the primers were empirically tested with fungal cultures and clinical samples. We provide data that these primers significantly improve fungal taxonomic coverage, fungal read recovery, and accuracy of characterization of human-associated fungal communities.

## RESULTS

### ITS1 primer design.

A total of seven (five forward and two reverse) published primers amplifying the ITS1 region were initially chosen for further analysis based on reported overall taxonomic coverage and amplicon size (see Table S1 in the supplemental material). Additionally, 85 primers (78 forward and 7 reverse) were custom designed using the SIS database (see Table S2 in the supplemental material). Three forward primers and one reverse primer representing the previously published primers and four forward primers and one reverse primer representing the custom primers were further evaluated by extended *in silico* testing using the SIS database and experimental testing of fungal cultures (Table 1). The four custom-designed forward primer pairs were also tested with one published reverse primer (Table 1). The chosen primer pairs were selected based on high taxonomic coverage of sequences in the UNITE (User-friendly Nordic ITS Ectomycorrhiza) database, in particular for medically relevant cervicovaginal taxa (not shown), as well as a <5°C difference in melting temperature (*T*_*m*_) between the forward and reverse primers.

10.1128/mSphere.00488-17.3 TABLE S1  Published primers initially selected for this study. *, a designation with “(L)” at the end of the name signifies that these primers were from the literature. Download TABLE S1, PDF file, 0.2 MB. Copyright © 2017 Usyk et al. 2017 Usyk et al. This content is distributed under the terms of the Creative Commons Attribution 4.0 International license.

10.1128/mSphere.00488-17.4 TABLE S2  Custom primers initially designed for this study. “(N)” after the primer name indicates these primers were newly designed. Download TABLE S2, PDF file, 0.1 MB. Copyright © 2017 Usyk et al. 2017 Usyk et al. This content is distributed under the terms of the Creative Commons Attribution 4.0 International license.

**TABLE 1:** Published and custom-designed primers used for *in silico* and experimental testing

Primer	Direction	Primer sequence (5′ to 3′)	Reference(s)
Published			
ITS1 (L)	Forward	TCCGTAGGTGAACCTGCGG	11
ITS1-F (L)	Forward	CTTGGTCATTTAGAGGAAGTAA	20–23
ITS5 (L)	Forward	GGAAGTAAAAGTCGTAACAAGG	11
ITS2 (L)^a^	Reverse	GCTGCGTTCTTCATCGATGC	11, 21, 23, 28–35
Custom designed			
ITS1-27F (N)	Forward	TACGTCCCTGCCCTTTGTAC	
ITS1-30F (N)	Forward	GTCCCTGCCCTTTGTACACA	
ITS1-34F (N)	Forward	CTGCCCTTTGTACACACCGC	
ITS1-48F (N)	Forward	ACACACCGCCCGTCGCTACT	
ITS1-217R (N)	Reverse	TTTCGCTGCGTTCTTCATCG	

a This published reverse primer was also tested against the four selected custom-designed forward primers. “(L)” designates primers from the literature, and "(N)" indicates primers newly designed for this study.

### *In silico* analyses.

**FIG 2:**
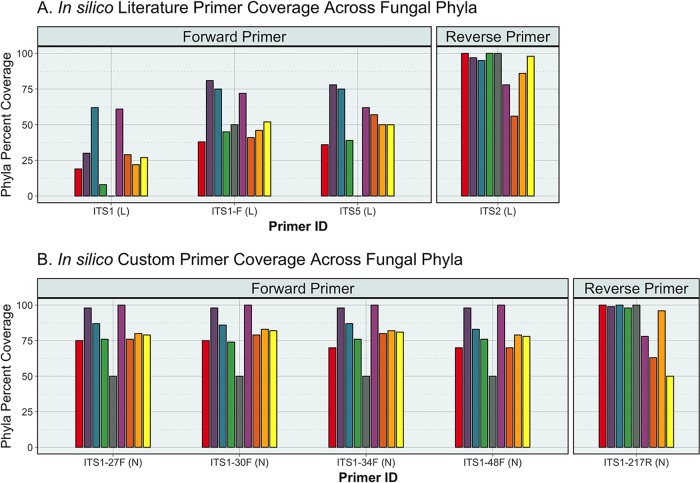
*In silico* coverage across fungal phyla. Predicted taxonomic coverage was assessed using PrimerProspector. (A) The forward literature fungal primers ITS1 (L), ITS1-F (L), and ITS5 (L) had significantly lower overall taxonomic coverage than the (B) newly created forward primers ITS1-27F (N), ITS1-30F (N), ITS1-34F (N), and ITS1-48F (N). The custom-designed reverse primer ITS1-217R (N) and the published reverse primer ITS2 (L) both demonstrated high predicted taxonomic coverage across the phyla.

**FIG 3:**
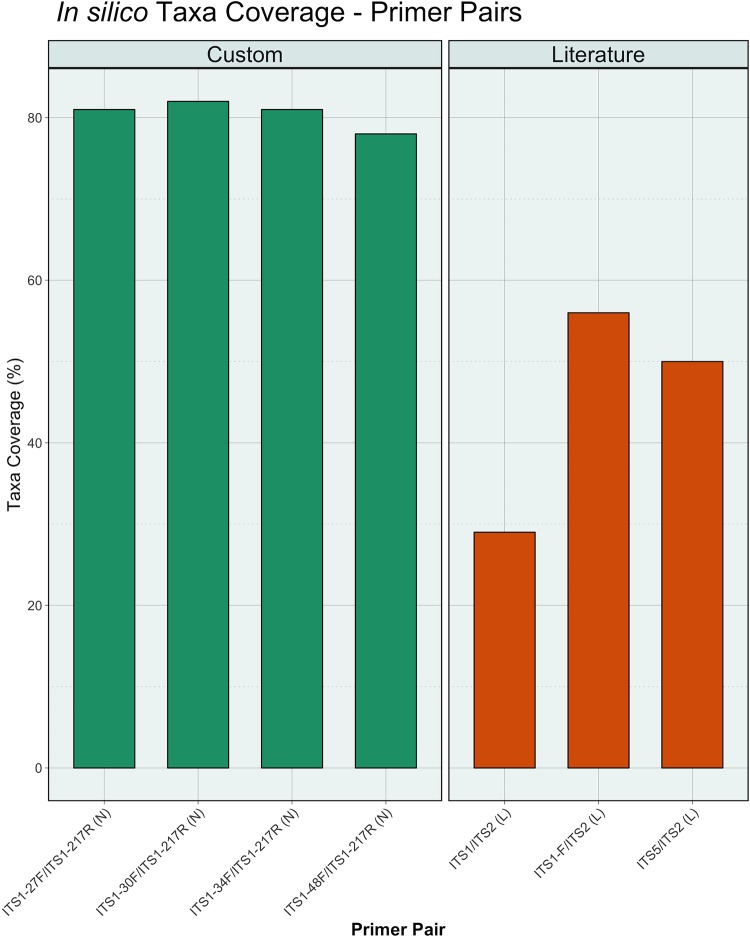
*In silico* taxon coverage. The average *in silico* taxonomic coverage estimates obtained with PrimerProspector for the selected forward primers from the literature [designated “(L)”] and newly designed forward primers [designated “(N)”]. The published ITS1 (L) primer was recommended by UNITE and is therefore shown in this figure despite the low coverage performance. Coverage estimates are based on 5,789 simulated species in the SIS database. Coverage estimates are based on the default alignment criteria of PrimerProspector.

*In silico* testing demonstrated that the custom-designed primers achieved an average *in silico* taxonomic coverage of 79.9% ± 7.1%, while the published primer pairs had an average coverage of 44.6% ± 13.2% (*P* = 0.05) (Fig. 2 and 3). Both reverse primers tested demonstrated similar performance, covering nearly all reference fungi, with coverage of 90.0% ± 14.8% and 87.1% ± 18.9% (*P* = 0.39) for the custom and published primers, respectively. This is likely due to the high conservation of the flanking 5.8S region (Fig. 1) compared to the larger and more variable 18S target of the forward primers. Coverage across the analyzed fungal phyla and *Candida* is summarized in Fig. S2 in the supplemental material. The custom primers were able to improve coverage of the medically relevant fungal genus *Candida* by being able to produce amplicons for all of its species contained in the SIS database compared to an average coverage of 59.5% ± 13% from the previously reported primers.

### Testing of fungal cultures.

**FIG 4:**
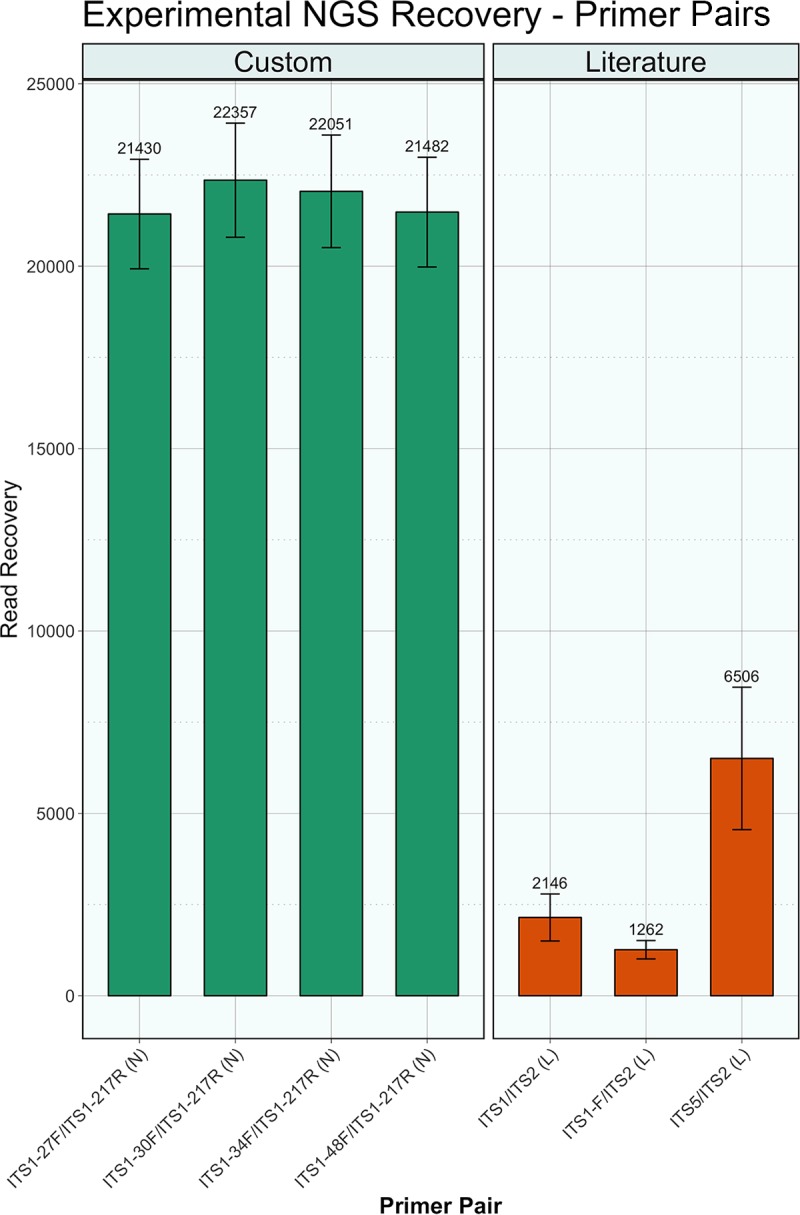
Experimental NGS read recovery. Average read recovery for the selected newly designed [designated “(N)”] and published forward primers from the literature [designated “(L)”] is shown at the top of each colored bar. Error bars show the standard error of the mean. The names of the primer pairs are indicated on the *x* axis.

The 13 fungal culture isolates used in this study represent common taxa found in the cervicovaginal tract and include species from two phyla (*Ascomycota* and *Basidiomycota*), six classes, and seven orders (see Table S3 in the supplemental material). Read recovery analysis for the selected primer pairs demonstrated that the custom-designed primer sets resulted in an order of magnitude increase over all but one published primer pair from the literature [designated by “(L)”]: there was a 3-fold increase over the ITS5 (L) primer (Fig. 4). The newly designed primers were able to achieve a mean sequencing depth of 21,830 ± 225 fungal reads, while the published primers amplified an average of 3,305 ± 1,621 fungal reads (*P* = 0.029). In addition to an increase in sequencing depth, there was a more consistent read recovery between the newly designed primer set [designated by “(N)”] than with the literature set. The 20 PCR-negative controls averaged 7 ± 2 fungal reads.

10.1128/mSphere.00488-17.5 TABLE S3  Number (and percentage) of sequences of each phyla in the UNITE database. Download TABLE S3, PDF file, 0.1 MB. Copyright © 2017 Usyk et al. 2017 Usyk et al. This content is distributed under the terms of the Creative Commons Attribution 4.0 International license.

Across the 13 fungal cultures (Table 2), the custom-designed primers provided an overall improvement approaching statistical significance (*P* = 0.057) in correct identification compared to the published primers. A culture was considered to be correctly identified if at least 50% of the reads from a given primer pair matched the species identification of the culture, the anamorph of the cultured species, or higher taxonomic strata that the cultured species belongs to. Custom primers were able to identify all cultures. The worst-performing primer was the published forward primer ITS1-F (L), which resulted in identification of 10 out of 13 cultures, while primers ITS1 (L) and ITS5 (L) both correctly identified 11 of the 13 cultures. This misidentification occurred for cultures when the primers had less than 1,000 reads, highlighting the importance of adequate sample sequencing depth.

**TABLE 2:** Fungal species tested in this study representing common human cervicovaginal-associated species

Laboratory ID	Species
Fungal_01	*Saccharomyces cerevisiae*
Fungal_02	*Rhodotorula mucilaginosa*
Fungal_15	*Candida dubliniensis*
Fungal_18	*Candida tropicalis*
Fungal_31	*Cryptococcus neoformans*
Fungal_33	*Aspergillus flavus*
Fungal_35	*Aspergillus niger*
Fungal_36	*Aspergillus terreus*
Fungal_37	*Bipolaris* sp.
Fungal_38	*Microsporum canis*
Fungal_39	*Scedosporium apiospermum*
Fungal_56	*Candida albicans*
Fungal_58	*Candida parapsilosis*

### Clinical sample analyses.

Reads from clinical samples were bioinformatically processed to remove any reads that were not identified as being fungal. Following this filtering, the clinical samples averaged 10,068 ± 1,227 fungal reads per sample. The read breakdown for each body site was as followed: the cervical samples had 6,221 ± 638 reads, whereas samples from the oral and anal sites had similar coverage of 11,357 ± 1,677 and 12,626 ± 3,174 fungal reads, respectively.

**FIG 5:**
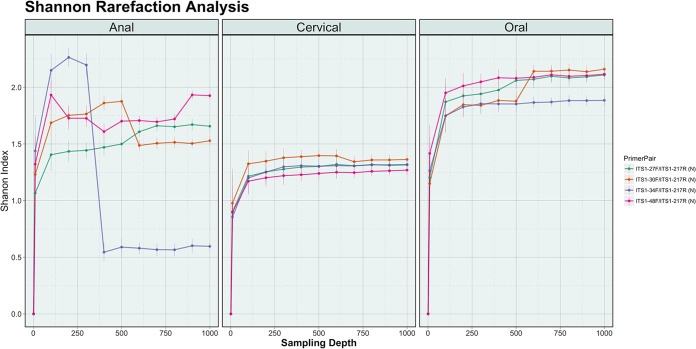
Shannon rarefaction analysis. Shown is Shannon alpha rarefaction analysis across three body sites for seven clinical samples using four newly designed primer pairs. Amplified samples were evaluated at depths of 1, 10, 100, 500, and 1,000 reads, with 100 replicates at each subsampling depth. Results for each primer pair with the samples were averaged and plotted by anatomic site. The primer pairs associated with each colored line are indicated in the key to the right of the figure.

Alpha rarefaction using the Shannon metric was used to determine whether the fungal community was adequately represented for each sample (Fig. 5). The majority of the sample-primer combinations achieved adequate sequencing depth across the three body sites: 74/84 achieved sufficient sampling depth with the plateau point being designated at 500 reads.

## DISCUSSION

Most mycobiome studies that utilize next-generation sequencing have typically used primer sets that were developed with a limited reference set of fungal species (11). Using *in silico* testing with a novel database (SIS), we determined that frequently cited primer sets rarely exceed 50% taxonomic coverage of fungal sequences in the UNITE database and result in low numbers of sequence reads during NGS runs (Fig. 3 and 4). As a result, these published primer sets limit our ability to adequately evaluate fungal communities. We determined using *in silico* testing and the newly designed SIS database that our newly designed primers are able to amplify 79.9% ± 7.1% of the species currently present in the UNITE database. In addition, we found using a sequencing experiment with fungal isolates that the redesigned primers increase fungal read recovery from 3,305 ± 1,621 reads to 21,830 ± 225 reads. We further show that the increased sequencing depth is such that it can capture the fungal diversity at three body sites. The only exception to this is the newly designed primer pair ITS1-34F/ITS1-217R (N), which failed for several samples in the anal region. The loss is likely due to technical or sequencing issues, as opposed to the limitation of the primers to amplify fungi from this anatomic region. A technical issue is more likely to be the case, rather than an inability of the custom primers to amplify anal fungi because nonmetric multidimensional scaling (NMDS) analysis of the fungal communities across the sites indicates that the anal region overlaps both the oral and cervical regions, meaning that the regions have common species of fungi present (see Fig. S1 in the supplemental material) that were detected with this primer pair.

10.1128/mSphere.00488-17.1 FIG S1  NMDS analysis of the fungal communities across three body sites. Clustering is based on Bray distance for three body sites plotted using NMDS. Ellipses represent a 95% confidence cluster area. Permutational multivariate analysis of variance (PERMANOVA) indicates that clustering due to body site explains 11.6% of the community variance (*P* < 0.001). Download FIG S1, TIF file, 2.2 MB. Copyright © 2017 Usyk et al. 2017 Usyk et al. This content is distributed under the terms of the Creative Commons Attribution 4.0 International license.

10.1128/mSphere.00488-17.2 FIG S2  Heat map showing the taxonomic coverage across the combined SIS fungal database. Heat map showing the taxonomic coverage across the 9 analyzed fungal phyla and *Candida* for each forward primer using the SIS database. Taxa are shown as rows, and forward primers are in the columns of the heat map. The name of the primer is given with “(L)” designating the literature primers and “(N)” designating the newly described primers. Download FIG S2, TIF file, 2.6 MB. Copyright © 2017 Usyk et al. 2017 Usyk et al. This content is distributed under the terms of the Creative Commons Attribution 4.0 International license.

The primers described in this report were designed using a custom database (SIS) that combined information within the UNITE (16) and SILVA (17) databases. The SIS database made it possible to perform *in silico* PCR testing because, to the best of our knowledge, there were no available databases that contain the entire 18S-ITS1-5.8S contig. The SIS database will be publically available to facilitate further public ITS amplification testing. Although we showed experimentally that the simulated database allows for the development of significantly better primers over the current primer sets, it should be noted that the availability of the true reference contigs would make taxonomic coverage more accurate. Nevertheless, use of *in silico* analyses may have unexpected limitations that can be elucidated on experimental testing.

The primers developed with the help of the SIS database significantly increased taxonomic coverage and increased NGS yield by an order of magnitude for fungal cultures. The increased recovery of fungal reads in the human samples was most notable in the cervicovaginal region, which is known for low microbial biomass (bacteria and fungi) compared to other human body sites (18). High read counts are crucial to ensure that fungal communities are accurately characterized. The best overall primer set for the study of the fungal mycobiome in the cervicovaginal region was the ITS1-30F/ITS1-217R (N) pair (Table 1). This primer pair was the highest performing in terms of overall fungal culture identification and the best performing in terms of fungal read recovery of all tested primers (Fig. 4), which is critical when studying a low-mycobiome-biomass site. It should be reiterated, however, that the differences between the new primer sets were marginal, and any of the four custom-designed pairs offers a significant improvement in read recovery and taxonomic coverage. These newly designed primers will facilitate fungal microbiome (mycobiome) studies by expanding taxonomic coverage and ensuring the accuracy of community characterization by providing significant improvements in fungal sequencing depth. This is particularly an important improvement for studies of fungal communities in the cervicovaginal body region, where these types of studies are currently lacking (19).

This study was limited by the lack of an actual intact ITS region database—thus the need to design a custom database (SIS) of the 18S-ITS-5.8S contig to facilitate *in silico* evaluation of custom fungal primers. Although the developed primers seem to reflect an improvement over the literature primers in experimental analyses, having a true 18S-ITS-5.8S database would increase the accuracy of the results. The study may also be limited by the current set of fungal reference sequences. With constant improvements made to sequencing technology and constant updates made to fungal reference databases, it may be necessary to reexamine the newly designed primer sets to ensure that they can adequately capture the ever-expanding fungal diversity and if necessary create new primer-sets.

This study demonstrates significant limitation of commonly used ITS primers and presents newly designed primers that significantly expand the general taxonomic coverage and increase NGS read recovery. Additionally, the study presents a full 18S-ITS-5.8S contig database (SIS) that can be used to evaluate the priming efficacy of the ITS region and offers suggested parameters for running postsequencing operational taxonomic unit (OTU) clustering analysis.

## MATERIALS AND METHODS

### ITS1 primer assessment and custom primer design.

Previously published ITS1 primers were identified from the literature [designated “(L)”], with an emphasis on studies of the human mycobiome and on fungal species of the terrestrial environment (Table S1) (11, 20–36). Additionally, primers recommended by the User-friendly Nordic ITS Ectomycorrhiza (UNITE) database were also considered (16). Primers from the literature and UNITE database were chosen for further investigation if they met the following criteria, based on published reports: (i) amplification of the ITS1 region, (ii) indication of utility as a general-purpose amplifier of the ITS1 locus without known large taxonomic biases, (iii) amplicon sizes below 700 bp and in which the predicted amplicon mean length for a given pair was 400 + 100 bp, and (iv) evidence of coverage of known human-associated fungal taxa.

To develop new custom forward primers [designated “(N)”], a 250-bp region from the 3′ end of the 18S fragment for each of the representative sequences in the SILVA high-quality rRNA database (17) was used to enable focus on the region immediately upstream of ITS1 (Fig. 1). These 18S subfragment sequences were aligned using MAFFT V7 (37). Of the 23,128 input sequences, some failed to align (*n =* 63) and were pruned from the input, and the entire set was realigned. The final aligned sequence set was then subjected to PrimerProspector (38) from the V1.9 release of QIIME virtualbox (39) to obtain degenerate forward primers (Table S2).

Reverse primers were created using the UNITE v7 database from the October 2014 release (10). The 5.8 S reference sequence fragment was selected in order to retrieve the contiguous ITS1 region with the flanking ribosomal sequences (Fig. 1). MAFFT v7 (37) was used for the alignment, and PrimerProspector was used to generate degenerate reverse primers. Due to the high conservation of the 5.8S region, no alignment pruning was needed.

### 18S-ITS1-5.8S (SIS) contig database creation.

To test primers *in silico* and estimate taxonomic coverage, a contig of the 18S-ITS1-5.8S region was required (Fig. 1); however, a specific database that covers the entire region from 18S to 5.8S does not to our knowledge exist, nor is this region included in the published genome sequences of most fungi. We therefore designed an algorithm to merge sequences from SILVA and UNITE databases to simulate an intact 18S-ITS1-5.8S region. The custom database was designed to include the ITS1 locus with the entire 18S 5′ region not just the targeted 250-bp 3′ fragment, in order to ensure that the designed primers did not anneal to multiple conserved targets within the 18S gene. Taxonomic levels within each database are not in the same format (i.e., SILVA has more taxonomic levels than UNITE, which is fixed at seven levels); therefore, an iterative regular expression pattern-matching algorithm (40) was used to match the taxonomic descriptor for each UNITE sequence to a SILVA reference at the lowest possible taxonomic level. This procedure took advantage of the high conservation of the ITS1 flanking rRNA loci, which make these regions suitable to resolve fungi to the species level (41). The newly combined SILVA-UNITE database, termed SIS (18S-ITS1-5.8S), made it possible to then perform *in silico* PCR testing since the forward and reverse primer targets were present on each contig. The SIS database contains 5,789 simulated reference sequence contigs representing 3,842 fungal species.

### *In silico* testing of primer sequences.

Published and custom-designed primers (Tables S1 and S2) were evaluated to identify pairs with melting temperature differences of <5°C using OligoCalc (42). Next, the primer pairs were scored for taxonomic coverage and amplicon size using the SIS database with PrimerProspector (39). The amplicon range was chosen to specifically work with the MiSeq platform and ranged from 358 ± 83 bp to 384 ± 105 bp. Finally, the top-performing published primers from the literature (3 forward and 1 reverse) and newly designed primers (4 forward and 1 reverse) in terms of global fungal coverage across all phyla found in the UNITE v7 database (Table S3) were selected for experimental *in silico* testing (Table 1). Additionally, the final primer pairs (Table 1) were chosen to include taxonomic coverage of fungi that have been previously found in the cervicovaginal region.

### Fungal cultures.

Thirteen fungal culture isolates were obtained from two sources—the College of American Pathologists (CAP) samples (*n =* 2) and from the microbiology lab at the Montefiore Medical Center (*n =* 11) (Table 2). The Montefiore samples were subcultured on appropriate media for growth of fungus and the Montefiore Microbiology Laboratory classified isolated colonies. Yeast organisms were identified using the Phoenix automated system for identification, and rice Tween agar was used to evaluate yeast morphology and confirm identification. Filamentous fungi were identified using standard mycology techniques (i.e., microscopy and temperature requirements).

### Clinical samples.

Samples from seven anonymous individuals from our published studies were used (43, 44). Cervicovaginal and anal swab samples were collected in PreservCyt (Thin Prep, Marlborough, MA), and Scope mouthwash was used for oral sample collection. Samples were transferred to a 15-ml tube and gently centrifuged at 1,500 rpm for 5 min. After removing the supernatant by decanting, the pellet was rinsed in 3 ml of TE (10 mM Tris, 1.0 mM EDTA). This solution was then vortexed and centrifuged at 1,500 rpm for 5 min, and the supernatant was removed by decanting. The remaining pellet and any leftover solution (~150 µl) were stored at −20°C until further processing.

### DNA extraction.

DNA was extracted from all fungal cultures and clinical samples in a sterile biosafety cabinet. Fungal culture samples were incubated at 37°C for 30 min in an enzyme cocktail containing 15 µl of lysozyme (0.84 mg/ml; Sigma-Aldrich), 9 µl of mutanolysin (0.25 U/ml; Sigma-Aldrich), and 4 µl lysostaphin (21.10 U/ml; Sigma-Aldrich). The samples were then incubated at 56°C for 10 min following the addition of 15 μl proteinase K (20 mg/ml) and 150 μl buffer AL (Qiagen lysis buffer) and mixed by pulse-vortexing for 15 s. The samples were transferred to a screw-cap tube with 100 mg of UV-sterilized 0.1-mm-diameter zirconia-silica beads (11079101z; BioSpec, Bartlesville, OK) and beaten with a FastPrep-24 homogenizer (MP Biomedicals, Santa Ana, CA) at speed 6 for 40 s. Tubes were centrifuged at 750 × *g* for 30 s, 150 µl of supernatant was removed and placed in a new tube, and then 100 µl of 100% ethanol (EtOH) was added and mixed by pulse-vortexing for 15 s. After centrifugation at 750 × *g* for 30 s, the supernatant was added to the QIAamp mini-spin column (Qiagen, Valencia, CA) and centrifuged at 6,000 × *g* for 1 min. Column purification was performed according to the QIAamp DNA minikit directions starting at the AWI wash step, and DNA was collected in 100 µl of buffer AE (10 mM Tris, 0.5 mM EDTA [pH 9]).

DNA was extracted from the clinical samples using the QIAamp mini-spin column method (Qiagen) following the manufacturer’s protocol. The purified DNA was eluted in 150 µl of elution buffer AE.

### PCR and sequencing.

The DNA from the fungal culture samples underwent PCR using three published primer pairs and eight primer pairs consisting of custom-designed plus published primers (Table 1). All primers were synthesized with Golay barcodes, providing unique dual barcodes for each PCR. For each primer pair, PCR protocols were optimized for annealing temperature using a Verti thermal cycler (Thermo Fisher, Waltham, MA). Based on these trials, PCR was performed in a 25-µl reaction mixture with 2.5 µl input of fungal culture DNA, 16.25 µl of double-distilled water (ddH_2_O), 2.5 µl of USB 10× buffer with MgCl_2_ (10 mM; Affymetrix, Santa Clara, CA), 1 µl of USB MgCl_2_ (25 mM), 0.5 µl of deoxynucleoside triphosphate (dNTP) mixture (10 mM each; Roche Basel, Switzerland), 0.25 µl AmpliTaq Gold polymerase (5 U/µl; Applied Biosystems, Carlsbad, CA), 0.5 µl of Hotstart-IT DNA Fidelitaq polymerase (2.5 U/µl; Affymetrix), and 1 µl (5 µM) of each primer (IDT, Coralville, IA). Thermocycling was performed on a GeneAmp PCR system 9700 (Applied Biosystems) and included an initial denaturation of 95°C for 3 min, followed by 35 cycles of 95°C for 30 s, 55°C for 30 s, and 68°C for 2 min, followed by a final extension of 68°C for 10 min. Negative controls were 20 mock samples with all reagents, including barcoded primers but without any extracted DNA that went through PCR and NGS.

PCRs of the clinical samples were performed using four custom-designed primer pairs (Table 1). For these samples, PCRs were performed as described above using 10 µl of sample DNA.

PCR products for fungal cultures and clinical samples were pooled, and 100 µl of the pooled PCR products was loaded into a 4% agarose gel and run at 88 V for 4 h until the bands separated. The band for each primer pair was excised, purified with a QIAquick gel extraction kit (Qiagen), and quantified using a Qubit 2.0 fluorometric high-sensitivity double-stranded DNA (dsDNA) assay (Life Technologies, Inc., Carlsbad, CA).

Next-generation sequencing library preparation was performed using the KAPA LTP library preparation kit (KAPA Biosystems, Wilmington, MA) according to the manufacturer’s protocol. The size integrity of the isolated amplicons was validated with a 2100 Bioanalyzer (Agilent Technologies, Santa Clara, CA) at the Genomics Core at Albert Einstein College of Medicine. High-throughput sequencing of libraries was carried out on an Illumina MiSeq (Illumina, San Diego, CA) with a 2× 300-bp paired-end read kit at the Genomics Core of the Albert Einstein College of Medicine.

### Bioinformatic pipeline and statistical analyses.

MiSeq sequence reads were demultiplexed based on the dual barcodes using Novocraft’s Novobarcode V1.00 (45). Reads were trimmed for bases that fell below a PHRED score of 25 at the 3′ end with PrinSeq V0.20.4 (46) and merged using PANDASEQ V1.20 (47) under the default settings.

Open reference OTU picking was employed using QIIME v1.9 open-reference OTU picking protocol and the UNITE database for the reference-based clustering component. VSEARCH v1.4.0 (48) was substituted for usearch within Mac Qiime to facilitate higher throughput, since VSEARCH allows all of a system’s available memory to be used for processing. The OTU clustering threshold was set at 99% sequence identity to account for fungal heterogeneity as previously reported (16). Sequence dereplication and chimera removal were performed using the QIIME quality control protocol. Representative sequences for each OTU cluster were chosen based on sequence abundance. BLAST (49) was used to assign taxonomy using the UNITE database. The default behavior of BLAST in QIIME was changed to a minimum of 99% sequence identity for taxonomic assignment.

Data were processed in R version 3.3.1 (50). QIIME outputs were imported into R using the *phyloseq* (51) package and further processed with *vegan* (52), *coin* (53), and *reshape2* (54). Data visualization was performed using *ggplot* (55). A Wilcoxon-Mann-Whitney test, *stats* package, was used for assessment of the number of sequence read counts and taxonomic coverage for fungal cultures amplified with custom-designed and previously published primers.

### Data availability.

Raw data from the UNITE and SILVA reference databases are available for download from the source websites. The constructed SIS database is available for public access through the associated GitHub page: https://github.com/musyk07/18S-ITS1-5.8S-SIS-Database. The algorithm used to construct the SIS database is also available on GitHub.
