# Supplementary material for: Novel ITS1 Fungal Primers for Characterization of the Mycobiome
Source: mSphere. 2017 Dec 13;2(6):e00488-17. doi: 10.1128/mSphere.00488-17 (PMC5729218; doi:10.1128/mSphere.00488-17)
Supplement: TABLE S1 [file sph006172425st3.pdf]

Table S1

**Published name***	**Direction**	**Primer sequence (5' to 3')**	**Reference(s)**
ITS1 (L)	Forward	TCCGTAGGTGAACCTGCGG	(1)
ITS1-F (L)	Forward	CTTGGTCATTTAGAGGAAGTAA	(2-5)
ITS5 (L)	Forward	GGAAGTAAAAGTCGTAACAAGG	(1)
ITSOF-T (L)	Forward	ACTTGGTCATTTAGAGGAAGT	(6, 7)
ITS1_F (L)	Forward	GTAAAAGTCGTAACAAGGTTTC	(8, 9)
ITS2 (L)	Reverse	GCTGCGTTCTTCATCGATGC	(1, 3, 5, 10-17)
FITS7R (L)	Reverse	CAAAGATTCGATGAYTCAC	(7)
ITS1_R (L)	Reverse	GTTCAAAGAYTCGATGATTCAC	(9)
58A2R (L)	Reverse	CTGCGTTCTTCATCGAT	(3, 18)

1. White TJ, Bruns T, Lee S, Taylor J. 1990. AmpliFication and direct sequencing oF Fungal ribosomal RNA genes For phylogenetics. PCR protocols: a guide to methods and applications 18:315-322.
2. Cui L, Morris A, Ghedin E. 2013. The human mycobiome in health and disease. Genome medicine 5:63.
3. Gardes M, Bruns TD. 1993. ITS primers with enhanced speciFicity For basidiomycetes ‐ application to the identiFication oF mycorrhizae and rusts. Molecular ecology 2:113-118.

4. Martin KJ, Rygiewicz PT. 2005. Fungal-speciFic PCR primers developed For analysis oF the ITS region oF environmental DNA extracts. BMC microbiology 5:28.

5. Ghannoum MA, Jurevic RJ, Mukherjee PK, Cui F, Sikaroodi M, Naqvi A, Gillevet PM. 2010. Characterization oF the oral Fungal microbiome (mycobiome) in healthy individuals. PLoS pathogens 6:e1000713.

6. Taylor DL, McCormick MK. 2008. Internal transcribed spacer primers and sequences For improved characterization oF basidiomycetous orchid mycorrhizas. New Phytologist 177:1020-1033.

7. Tedersoo L, Anslan S, Bahram M, Põlme S, Riit T, Liiv I, Kõljalg U, Kisand V, Nilsson H, Hildebrand F. 2015. Shotgun metagenomes and multiple primer pair-barcode combinations oF amplicons reveal biases in metabarcoding analyses oF Fungi. MycoKeys 10:1.

8. Lennon NJ, Lintner RE, Anderson S, Alvarez P, Barry A, Brockman W, Daza R, Erlich RL, Giannoukos G, Green L. 2010. A scalable, Fully automated process For construction oF sequence-ready barcoded libraries For 454. Genome biology 11:R15.

9. Willger SD, Grim SL, Dolben EL, Shipunova A, Hampton TH, Morrison HG, Filkins LM, George AT, Moulton LA, Ashare A. 2014. Characterization and quantiFication oF the Fungal microbiome in serial samples From individuals with cystic Fibrosis. Microbiome 2:40.

10. De Beeck MO, Lievens B, Busschaert P, Declerck S, Vangronsveld J, Colpaert JV. 2014. Comparison and validation oF some ITS primer pairs useFul For Fungal metabarcoding studies. PLoS One 9:e97629.

11. Drell T, Lillsaar T, Tummeleht L, Simm J, Aaspõllu A, Väin E, Saarma I, Salumets A, Donders GG, Metsis M. 2013. Characterization oF the vaginal micro-and mycobiome in asymptomatic reproductive-age Estonian women. PloS one 8:e54379.

12. Dupuy AK, David MS, Li L, Heider TN, Peterson JD, Montano EA, Dongari-Bagtzoglou A, Diaz PI, Strausbaugh LD. 2014. RedeFining the human oral mycobiome with improved practices in amplicon-based taxonomy: discovery oF Malassezia as a prominent commensal. PLoS One 9:e90899.

13. HoFFmann C, Dollive S, Grunberg S, Chen J, Li H, Wu GD, Lewis JD, Bushman FD. 2013. Archaea and Fungi oF the human gut microbiome: correlations with diet and bacterial residents. PloS one 8:e66019.

14. Mukherjee PK, Chandra J, Retuerto M, Sikaroodi M, Brown RE, Jurevic R, Salata RA, Lederman MM, Gillevet PM, Ghannoum MA. 2014. Oral mycobiome analysis oF HIV-inFected patients: identiFication oF Pichia as an antagonist oF opportunistic Fungi. PLoS pathogens 10:e1003996.

15. Orgiazzi A, Bianciotto V, BonFante P, Daghino S, Ghignone S, Lazzari A, Lumini E, Mello A, Napoli C, Perotto S. 2013. 454 pyrosequencing analysis oF Fungal assemblages From geographically distant, disparate soils reveals spatial patterning and a core mycobiome. Diversity 5:73-98.

16. Tang J, Iliev ID, Brown J, Underhill DM, Funari VA. 2015. Mycobiome: approaches to analysis oF intestinal Fungi. Journal oF immunological methods 421:112-121.
17. Bellemain E, Carlsen T, Brochmann C, Coissac E, Taberlet P, Kauserud H. 2010. ITS as an environmental DNA barcode For Fungi: an in silico approach reveals potential PCR biases. BMC microbiology 10:189.
18. Sapkota R, Knorr K, Jørgensen LN, O'Hanlon KA, Nicolaisen M. 2015. Host genotype is an important determinant oF the cereal phyllosphere mycobiome. New Phytologist 207:1134-1144.
