# Supplementary material for: Novel ITS1 Fungal Primers for Characterization of the Mycobiome
Source: mSphere. 2017 Dec 13;2(6):e00488-17. doi: 10.1128/mSphere.00488-17 (PMC5729218; doi:10.1128/mSphere.00488-17)
Supplement: TABLE S2 [file sph006172425st4.pdf]

Table S2

Forward primers	Primer sequence (5' to 3')	Reverse primers	Primer sequence (5' to 3')
ITS1-16F (N)	CTYGCGTTGATTACGTCCCT	ITS1-197R (N)	ATGCCAGAACCAAGAGATCC
ITS1-30F (N)	GTCCCTGCCCTTTGTACACA	ITS1-217R (N)	TTTCGCTGCGTTCTTCATCG
ITS1-12F (N)	TCAGCTYGCGTTGATTACGT	ITS1-219R (N)	CATTTCGCTGCGTTCTTCAT
ITS1-54F (N)	CCGTCGCTACTACCGATTGA	ITS1-222R (N)	TCGCATTTCGCTGCGTTCTT
ITS1-31F (N)	TCCCTGCCCTTTGTACACAC	ITS1-252R (N)	TTCACTGAATTCTGCAATTC
ITS1-29F (N)	CGTCCCTGCCCTTTGTACAC	ITS1-281R (N)	GGCGCAATGTGCGTTCAAAG
ITS1-13F (N)	CAGCTYGCGTTGATTACGTC	ITS1-282R (N)	GGGCGCAATGTGCGTTCAAA
ITS1-15F (N)	GCTYGCGTTGATTACGTCCC		
ITS1-14F (N)	AGCTYGCGTTGATTACGTCC		
ITS1-28F (N)	ACGTCCCTGCCCTTTGTACA		
ITS1-53F (N)	CCCGTCGCTACTACCGATTG		
ITS1-39F (N)	CTTTGTACACACCGCCCGTC		
ITS1-51F (N)	CGCCCGTCGCTACTACCGAT		
ITS1-52F (N)	GCCCGTCGCTACTACCGATT		
ITS1-32F (N)	CCCTGCCCTTTGTACACACC		
ITS1-33F (N)	CCTGCCCTTTGTACACACCG		
ITS1-27F (N)	TACGTCCCTGCCCTTTGTAC		
ITS1-26F (N)	TTACGTCCCTGCCCTTTGTA		
ITS1-25F (N)	ATTACGTCCCTGCCCTTTGT		
ITS1-23F (N)	TGATTACGTCCCTGCCCTTT		

ITS1-50F (N)	CCGCCCGTCGCTACTACCGA
ITS1-49F (N)	ACCGCCCGTCGCTACTACCG
ITS1-17F (N)	TYGCGTTGATTACGTCCCTG
ITS1-11F (N)	ATCAGCTYGCGTTGATTACG
ITS1-43F (N)	GTACACACCGCCCGTCGCTA
ITS1-40F (N)	TTTGTACACACCGCCCGTCG
ITS1-35F (N)	TGCCCTTTGTACACACCGCC
ITS1-57F (N)	TCGCTACTACCGATTGAATG
ITS1-34F (N)	CTGCCCTTTGTACACACCGC
ITS1-58F (N)	CGCTACTACCGATTGAATGG
ITS1-56F (N)	GTCGCTACTACCGATTGAAT
ITS1-55F (N)	CGTCGCTACTACCGATTGAA
ITS1-10F (N)	CATCAGCTYGCGTTGATTAC
ITS1-24F (N)	GATTACGTCCCTGCCCTTTG
ITS1-22F (N)	TTGATTACGTCCCTGCCCTT
ITS1-42F (N)	TGTACACACCGCCCGTCGCT
ITS1-18F (N)	YGCGTTGATTACGTCCCTGC
ITS1-37F (N)	CCCTTTGTACACACCGCCCG
ITS1-19F (N)	GCGTTGATTACGTCCCTGCC
ITS1-41F (N)	TTGTACACACCGCCCGTCGC
ITS1-36F (N)	GCCCTTTGTACACACCGCCC
ITS1-38F (N)	CCTTTGTACACACCGCCCGT
ITS1-137F (N)	GYTGKTCAAACTYGGTCATT

ITS1-9F (N)	TCATCAGCTYGCGTTGATTA
ITS1-67F (N)	CGATTGAATGGCTYAGTGAG
ITS1-20F (N)	CGTTGATTACGTCCCTGCCC
ITS1-139F (N)	TGKTCAAACTTGGTCATTTA
ITS1-136F (N)	AGYTGGTCAAACTTGGTCAT
ITS1-49F (N)	CACACCGCCCGTCGCTACTA
ITS1-50F (N)	ACACCGCCCGTCGCTACTAC
ITS1-21F (N)	GTTGATTACGTCCCTGCCCT
ITS1-66F (N)	CCGATTGAATGGCTYAGTGA
ITS1-60F (N)	CTACTACCGATTGAATGGCT
ITS1-143F (N)	CAAACTTGGTCATTTAGAGG
ITS1-140F (N)	GGTCAAACTTGGTCATTTAG
ITS1-141F (N)	GTCAAACTTGGTCATTTAGA
ITS1-135F (N)	AAGYTGGTCAAACTTGGTCA
ITS1-144F (N)	AAACTTGGTCATTTAGAGGA
ITS1-51F (N)	CACCGCCCGTCGCTACTACC
ITS1-59F (N)	GCTACTACCGATTGAATGGC
ITS1-142F (N)	TCAAACTTGGTCATTTAGAG
ITS1-138F (N)	YTGDTCAAACTYGGTCATTT
ITS1-4F (N)	GCRAGTCATCAGCTYGCGTT
ITS1-6F (N)	RAGTCATCAGCTYGCGTTGA
ITS1-5F (N)	YRAGTCATCAGCTYGCGTTG
ITS1-47F (N)	TACACACCGCCCGTCGCTAC

ITS1-48F (N)	ACACACCGCCCGTCGCTACT
ITS1-129F (N)	GCYGRRAAGYTGRTCAAACT
ITS1-145F (N)	AACTTGGTCATTTAGAGGAA
ITS1-8F (N)	GTCATCAGCTYGCGTTGATT
ITS1-128F (N)	DGCYGRRAAGYTGRTCAAAC
ITS1-7F (N)	AGTCATCAGCTYGCGTTGAT
ITS1-157F (N)	TAGAGGAAGTAAAAGTCGTA
ITS1-154F (N)	ATTTAGAGGAAGTAAAAGTC
ITS1-158F (N)	AGAGGAAGTAAAAGTCGTAA
ITS1-160F (N)	AGGAAGTAAAAGTCGTAACA
ITS1-161F (N)	GGAAGTAAAAGTCGTAACAA
ITS1-130F (N)	CBGARAAKYTGRTCAAACTT
