# Supplementary material for: Novel ITS1 Fungal Primers for Characterization of the Mycobiome
Source: mSphere. 2017 Dec 13;2(6):e00488-17. doi: 10.1128/mSphere.00488-17 (PMC5729218; doi:10.1128/mSphere.00488-17)
Supplement: TABLE S3 [file sph006172425st5.pdf]

Table S3

**Phyla**	**Number of sequences**	**Percent**
Ascomycota	11321	48.95%
Basidiomycota	10039	43.41%
Cercozoa	5	0.02%
Chytridiomycota	146	0.63%
Ciliophora	1	0.00%
Glomeromycota	768	3.32%
Incertae sedis	6	0.03%
Neocallimastigomycota	1	0.00%
Rozellomycota	27	0.12%
Zygomycota	438	1.89%
Unidentified	376	1.63%
