# Supplementary figures and images for: Novel ITS1 Fungal Primers for Characterization of the Mycobiome
Source: mSphere. 2017 Dec 13;2(6):e00488-17. doi: 10.1128/mSphere.00488-17 (PMC5729218; doi:10.1128/mSphere.00488-17)

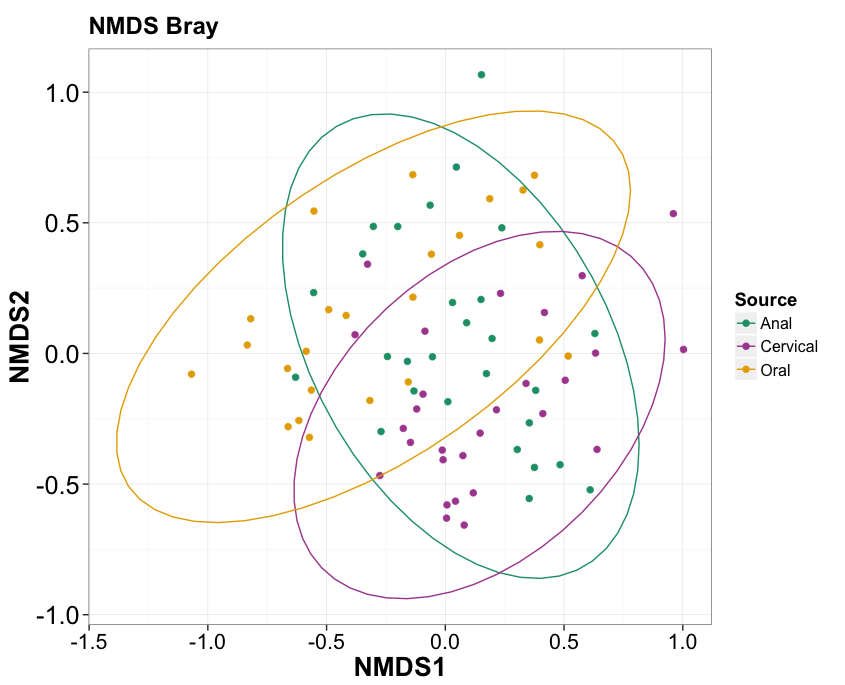

Supplement: FIG S1 [file sph006172425sf1.tif]

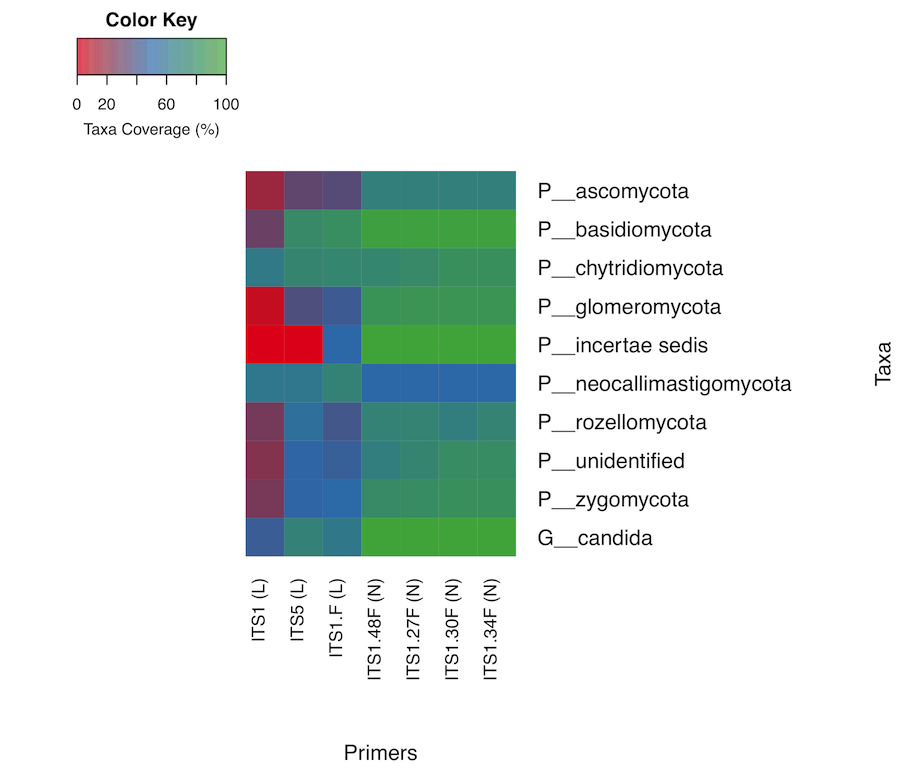

Supplement: FIG S2 [file sph006172425sf2.tif]
